# Reply to Henschel et al. Comment on “Qiu et al. Effect of Protein-Rich Breakfast on Subsequent Energy Intake and Subjective Appetite in Children and Adolescents: Systematic Review and Meta–Analysis of Randomized Controlled Trials. *Nutrients* 2021, *13*, 2840”

**DOI:** 10.3390/nu15071656

**Published:** 2023-03-29

**Authors:** Meijuan Qiu, Yu Zhang, Yuna He

**Affiliations:** 1 National Institute for Nutrition and Health, Chinese Center for Disease Control and Prevention, No. 29 Nanwei Road, Xicheng District, Beijing 100050, China; 2 Department of Clinical Nutrition and Department of Health Medicine, Peking Union Medical College Hospital, Chinese Academy of Medical Sciences (CAMS) and Peking Union Medical College (PUMC), No. 1 Shuaifuyuan, Dongcheng District, Beijing 100730, China

We want to thank Henschel et al. [1] for their interest and insightful comment on our paper [2]. After reviewing the comment, we would like to reply to the points they made one by one.

Firstly, we have corrected the data extraction errors mentioned in the comment, checked all the data and analyses again, and updated our original paper. Secondly, in the comment, Henschel et al. offered two statistical concerns and solutions: (1) calculating standard deviations of the treatment effect in crossover studies; (2) combining multiple treatment/control diets from the same study. We noticed that the result of the subsequent energy intake between the protein-rich and control breakfast exactly changed from “−111.2 [−147.6; −72.8]” to “−106.8[−130.3; −83.2]”. However, our main conclusion does not change for the association of breakfast protein content with subsequent energy intake. We finally discussed and decided not to revise the statistical method. The reasons are described as follows.

(1) Although our selection criteria were summarized through a large amount of related material, the studies ultimately included are still affected by a multitude of factors that result in large heterogeneity among studies. We had noticed this as well, and we had discussed the methodological differences and the large heterogeneity in our original paper. (2) Regarding the correlation coefficients in crossover studies and the multi-arm trials in the included studies, different researchers deal with it in different ways. In the comment, it was mentioned that “the Cochrane Handbook recommends either combining groups/conditions into a single comparison (recommended), omitting groups not relevant for the comparison, adjusting the sample size in the shared group, or conducting a network meta-analysis”. The statistical problem has a variety of solutions. For example, Sievert et al. [1] adopted a rather conservative approach, assumed a correlation coefficient of 0.3, and conducted sensitivity analyses with the following correlation coefficients: 0.5, 0.7, and 0.9, and ended up with robust results. Moreover, they divided the control group sample size in half in the multi-arm trials. trials. (3) The traditional statistical analysis method we adopted could also obtain a trustworthy result to an extent, although it has some defects in statistics. Although our statistical methods are not the most convincing, there are many studies using this traditional analysis method [3,4,5,6,7,8]. In addition, we adopted a plot digitizer software extracted the partial results regarding the subjective appetite components. These estimated values were generally in agreement with the actual values, with some deviation, and we conducted the traditional analyses to ensure the consistency of the statistical methods. (4) We admit that the method of statistical analysis suggested in the comment is a better way to address the concerns of the authors. Nevertheless, while theoretical methods and statistical analyses are continuously improving, there are still some bottlenecks. Compared with the better statistical methods, we paid more attention to the clinical and public health significance. More attention should be paid to statistical matters in the future.

Therefore, we had added “Five, the study design of the included studies affects the weight of each study in the odds ratio. In theory, the crossover studies would have more impact than the parallel study, as ignoring within-person variation. And the multiarm trials in the included studies [9,10,11,12] would also affect the overall result. Further studies are required to address these issues in the future.” to the limitation.

Finally, we are very sorry for our mistake, and we are very regretful that we discussed but ultimately decided not to revise the statistical method.
